# Impact of Endurance Exercise Training on Biomarkers of Aortic Endothelial Damage in Diabetic Rats

**DOI:** 10.1155/2024/6025911

**Published:** 2024-07-09

**Authors:** Mahtab Fouladi, Maryam Mahmoudabady, Zahra Gholamnezhad, Sadegh Shabab, Saeed Niazmand, Hossein Salmani

**Affiliations:** ^1^ Department of Physiology Faculty of Medicine Mashhad University of Medical Sciences, Mashhad, Iran; ^2^ Applied Biomedical Research Center Mashhad University of Medical Sciences, Mashhad, Iran; ^3^ Department of Physiology and Pharmacology Faculty of Medicine Sabzevar University of Medical Sciences, Sabzevar, Iran

**Keywords:** diabetes, exercise, metformin, oxidative stress, vascular endothelium

## Abstract

Given the heightened risk of diabetes-related cardiovascular events associated with inactivity, this study investigates the molecular mechanisms of vascular damage in streptozotocin (STZ)-induced diabetic rats. The aim is to elucidate the impact of different exercises (interval and continuous training) and metformin on biochemical parameters, aortic injury, oxidative stress, and inflammation to provide insights into potential therapeutic interventions for diabetes-associated vascular complications. Male Wistar rats were administered a single dose of STZ (60 mg/kg) to induce diabetes. Diabetic rats underwent either interval training or continuous training (40 min/day, 5 days/week, 6 weeks), received metformin (300 mg/kg), or a combination of metformin and exercise. After 6 weeks, biochemical parameters in serum and oxidative stress markers and mRNA expression of endothelial nitric oxide synthase (eNOS), lectin-like oxidized low-density lipoprotein receptor-1 (LOX-1), and intercellular adhesion molecule-1 (ICAM-1) in aorta tissue were assessed. Serum levels of fasting blood sugar (FBS), triglyceride (TG), total cholesterol (TC), low-density lipoprotein (LDL), TG/HDL, TC/HDL, and LDL/HDL ratios were significantly reduced in all treatment groups compared to the diabetes group. Both types of exercises, metformin, and exercise+metformin combinations, significantly reduced oxidative stress by decreasing malondialdehyde (MDA) and enhancing the antioxidant status in the aortic tissue compared to the diabetic group. In addition, in exercise groups, metformin, and combination groups, the expression of eNOS was significantly elevated, while LOX-1 and ICAM-1 expression significantly decreased compared to the diabetic group. In most cases, the combination of exercise and metformin (especially interval training) was more effective than exercise alone. It seems that exercise along with taking metformin can be considered as a therapeutic method by improving hyperglycemia and hyperlipidemia and reducing oxidative stress and vascular inflammatory responses, leading to ameliorating biomarkers function related to endothelial damage in experimental diabetes conditions.

Summary

• Interval and continuous training improved vascular health markers
• Combined use of metformin with exercise enhanced endothelial function
• Exercise and metformin prevented oxidative stress injury to endothelial tissue

## 1. Introduction

A sedentary lifestyle and obesity are highly related to the progressive prevalence of Type 2 diabetes mellitus (T2DM), which is associated with hyperglycemia and hyperlipidemia in diabetic patients [1]. The prevalence of diabetes, according to the International Diabetes Federation report, was 537 million adults in 2021, and this number will increase to 643 million by 2030 [2]. Diabetes mellitus (DM) is one of the major risk factors for the development of vascular complications, which increase the rate of morbidity and mortality in DM patients [3, 4]. Endothelial dysfunction and vascular injury in diabetes are caused by hyperglycemia, hyperlipidemia, oxidative stress, and inflammation [5]. Two well-known pathogenic features of T2DM that substantially impair vascular function are hyperglycemia and insulin resistance [6]. Vasoconstriction, leukocyte adherence, vascular inflammation, and thrombosis are all symptoms of atherosclerosis and endothelial dysfunction, which are characterized by a decrease in the bioavailability of vasodilators, a rise in endothelium-derived contracting factors and augmented expression of chemokines, cell adhesion molecules, cytokines, and the endothelial generation of reactive oxygen species (ROS) [7, 8]. Dyslipidemia and an increase in low-density lipoprotein (LDL) to high-density lipoprotein (HDL) ratio are usually regarded as risk factors for increasing endothelial dysfunction, a key element in the development of atherosclerosis [9]. Hyperglycemia triggers the overproduction of ROS, which in turn, through several pathways, could exert its detrimental effects, such as lipid peroxidation and the production of highly reactive aldehydes such as malondialdehyde (MDA), destruction of antioxidant defense systems including glutathione peroxidase (GPx), superoxide dismutase (SOD) and catalase (CAT) [10, 11]. Nitric oxide (NO), in addition to being a potent vasodilator, prevents the proliferation of vascular smooth muscle cells and the production of proinflammatory factors and adhesion molecules [12]. Diabetes decreases the activity of endothelial nitric oxide synthase (eNOS) and increases ROS generation, resulting in a decrease in NO bioavailability and enhancement of proatherogenic changes [13]. Increased production of ROS induces oxidized low-density lipoprotein (ox-LDL) formation [4]. Ox-LDL, by activating the (LOX-1), subsequently causes the development of vascular complications and atherosclerosis by triggering inflammatory responses and the deposition of lipids in susceptible vessels [4]. In the early stages, leukocyte adherence and transendothelial migration play a critical role in vascular injury and atherogenesis. Various adhesion molecules, such as intercellular adhesion molecule 1 (ICAM-1) and vascular cell adhesion molecule 1 (VCAM-1), participate in these processes [14]. The plasma level of adhesion molecules is elevated in individuals with atherosclerosis and patients with DM [15].

According to previous reports, people with an active lifestyle are less likely to develop insulin resistance and impaired glucose tolerance [16]. Aerobic exercise training has consistently been recommended as a cardiovascular protector for T2DM [3]. A study on diabetic and prediabetic patients revealed that high and moderate-intensity exercise can reduce fasting blood glucose [17]. Regular aerobic exercise ameliorates diabetes-induced cardiovascular complications such as hypertension, hyperlipidemia, and atherosclerosis. Exercise training has been proven to alleviate diabetic endothelial dysfunction by controlling hyperglycemia, which reduces the formation of free radicals and oxidative stress, resulting in augmentation of eNOS activity and NO production in the vascular system [18, 19].

In addition, exercise might reduce vascular endothelial damage by lowering plasma LDL-C, ox-LDL, and LOX-1 gene expression [18]. Moreover, exercise as a nonpharmacological method improves vascular inflammation and reduces the expression of factors involved in inflammation, including ICAM-1 and VCAM-1, ultimately preventing the progression of atherosclerosis [14, 20]. The beneficial effects of exercise on vascular function vary based on the intensity, type (resistance or endurance), and duration of training. Although in patients at risk of cardiovascular disease, high-intensity interval training (HIIT) is a more potent stimulus for improving vascular function (brachial artery flow-mediated dilation) than moderate-intensity continuous training (MICT). However, there is insufficient evidence about the best exercise prescription for diabetic patients [12].

Moreover, the biological mechanisms of exercise's protective role against diabetes-induced vascular complications should be more elucidated. Metformin, as a first-line treatment for diabetes, controls glucose levels and improves cardiovascular function in patients with diabetes and cardiovascular problems [21]. To our knowledge, few studies have been conducted to compare exercise with different types and investigate the effect of drugs such as metformin and physical activity. Therefore, in this study, we aimed to investigate and compare the effects of interval and continuous training (Cont) and metformin on the biological factors involved in endothelial damage in streptozotocin (STZ)-induced diabetic rats. However, STZ-induced diabetes is a well-established model for investigating type 1 DM. Experimental studies use it to evaluate the cardiovascular complications of diabetes in T2D because the physiopathological aspects of this model match the histopathological and metabolic changes associated with cardiomyopathy and typical DM2 cardiovascular disease [22–24].

## 2. Materials and Methods

### 2.1. Animals

Fifty-six male Wistar rats (250 ± 20 gr weight, 10 weeks old) were delivered from the Animal Care Center of Mashhad Medical School. The rats were kept in standard conditions (temperature 20 ± 2°C, 12-h light/dark cycle) with free access to water and the standard diet of rodents. The research was carried out in line with the Ethics Committee of Mashhad University of Medical Sciences for experimental animal use and care (IR.MUMS.MEDICAL.REC.1400.068; 2021-01-26). Animals were maintained in a lab environment for 1 week before the beginning of the study to adapt to experimental conditions.

### 2.2. Experimental Design

To induce diabetes in rats, after 8 h of fasting, they received a single dose of STZ (60 mg/kg, ip) [24]. In the control group, an STZ vehicle (sodium chloride 0.9%, 1 ml/kg ip) was injected. To assess fasting blood sugar (FBS), blood samples were taken from rats' tails 72 h after the STZ injection. Animals with more than 250 mg/dl of fasting blood glucose were selected as diabetic samples [25]. Diabetic animals were randomly put into seven groups (7 rats in each group), and their classification is shown in Table 1. Upon the study protocol, rats in different groups underwent treatment with metformin (300 mg/kg by gavage daily) [24, 26] and various types of exercise daily, 5 days/week for 6 weeks. Animals in the control and diabetes groups received equal saline as the vehicle. The day after the last session of training, after 8 h of fasting, the rats were anesthetized profoundly with ketamine-xylazine (60 mg/kg and 8 mg/kg, respectively, ip) [24]. After opening the chest, blood was collected from the heart for biochemical analysis. The thoracic and abdominal aortic tissue was quickly harvested, and a part of it was kept in RNAlater (Zist Kavosh Iranian Company, IRAN) for gene expression assessment. The remaining was conserved at −80 °C for oxidative stress measurement. Brain tissue was also removed to evaluate parameters related to memory and behavior in another study [27].

### 2.3. Exercise Training Protocol

**Figure 1:**
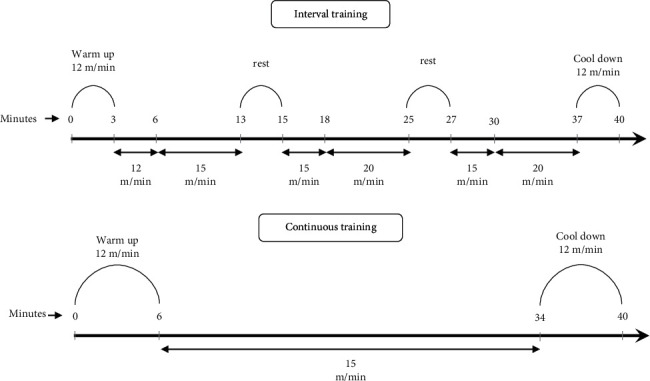
The protocol of interval training (Int) and continuous training (Cont).

A motorized treadmill was used to perform training protocols. In the familiarizing week, diabetic animals were assessed for their ability to exercise training. Briefly, animals were trained at a speed of 12 m/min at a 0%-degree inclination, 10 min/day, for 5 days, then were assessed and scored 1–5 based on running quality. The rats with a mean rating score of 3 or higher were included in the study [28]. Eligible animals were randomly divided into six groups (*n* = 8). The rats in the exercise groups were adapted to a progressive load of training (40 min/day, 5 days/week, for 6 weeks) to achieve the best performance and cardiorespiratory fitness and were trained as follows: Cont at a speed of 15 m/min [29] and interval training (Int) at a speed of 20 m/min [30] with 0% degree inclination were performed for animal training. The first 3 min of the training session were dedicated to warming up, and the last 3 min to cooling down (Figure 1). The intensity of animal training programs (Cont and Int) was evaluated at the end of the first, third, and fifth weeks [31], which indicated that the training load was 50%–70% VO_2_ max. The rats of the sedentary groups (control, diabetes, and metformin) were placed on the immobile treadmill to acclimate to the treadmill's environmental stresses.

### 2.4. Biochemical Assays

Fasting blood glucose was evaluated three times during the study: before STZ injection and 3 days after STZ injection by glucometer using a tail blood sample. Also, the heart-drained blood sample was used for FBS analysis (Cat No: 117500) and lipid profile assessment.

Lipid profile including triglyceride (TG) (Cat No: 132500), total cholesterol (TC) (Cat No: 110500), LDL-cholesterol (Cat No: 123050), and HDL-cholesterol (Cat No: 312050), were measured using the enzymatic photometric method. All measurements were made according to the manufacturer's instructions (Pars Azmoon Co., Tehran, Iran).

In addition, the ratios of TG/HDL, TC/HDL, and LDL/HDL were calculated using the measured amounts of these parameters and considered atherogenic indices [32].

### 2.5. Oxidative Stress Biomarker Measurement

The aorta samples (100 mg) were homogenized with an ultrasound homogenizer in phosphate buffer (pH 7.4). Then, the homogenates were centrifuged at 5000 rpm for 10 min at 4 °C. The supernatants were collected and used to evaluate oxidative stress markers; MDA, thiol groups, SOD, and CAT activity.

The MDA level measurement was used to determine the lipid peroxidation of aortic tissue. MDA creates a pink-colored complex when it interacts with thiobarbituric acid (TBA), with a peak absorbance of 535 nm [33]. To measure the MDA level, 1 mL of homogenized aortic tissue was mixed with 2 mL of a mixture solution containing hydrogen chloride, TBA, and trichloroacetic acid (HCL/TBA/TCA). The mentioned mixture was boiled for 40 min. Then, the samples were cooled and centrifuged for 10 min. The sample absorbance was calculated at 535 nm [34]. MDA level was calculated based on the following formula [35]: *C* (*M*) = *A*/1.65 × 10^5^.

As a total thiol indicator in the aorta, 2,2′-dinitro-5,5′-dithiodibenzoic acid (DTNB) interacts with the SH (thiol) groups to generate a yellow-colored solution. In this procedure, 50 *μ*L of aortic homogenized tissue was added to a solution containing tris and ethylenediaminetetraacetic acid (EDTA) buffer (pH = 8.6). The absorbance of the solution was then read at 412 nm (A1). Then, the DTNB reagent (20 *μ*L, 10 mM) was mixed with the solution, and the samples were stored for 15 min at room temperature (25°C), and the absorbance was read again (A2). The absorbance of the DTNB solution was also read and considered a blank (B). The thiol concentration was computed using the following formula [34, 36]: Total thiol concentration mM = (*A*2 − *A*1 − *B*) × 1.07/0.05 × 13.6.

The determination of SOD activity in aorta tissue was performed by the colorimetric method of Madesh and Balasubramanian [37]. The procedure involving the generation of superoxide through autooxidation of pyrogallol and the inhibition of superoxide-dependent reduction of the tetrazolium dye, MTT (3-(4, 5-dimethylthiazol-2-yl) 2, 5- diphenyltetrazolium bromide) conversion to formazan. The homogenized aorta tissue was pipetted into 96-well plates and incubated at room temperature (25°C) for 5 min. The reaction was stopped by adding dimethyl sulfoxide (DMSO), and then SOD activity was measured at 570 nm. One unit of SOD was defined as the amount of protein required to inhibit the rate of MTT reduction by 50%. The SOD activity was investigated at 570 nm and expressed as unit tissue [37].

The Aebi method was used to determine CAT activity. Hydrogen peroxide was utilized as the substrate in this procedure. The substrate used was 30 mM hydrogen peroxide (H2O2), and the substrate in control was 50 mM phosphate buffer (pH = 7). H2O2 was added to start the reaction, and for 3 min, the drop in absorption was monitored at 240 nm [38].

### 2.6. Gene Expression Measurement

#### 2.6.1. RNA Extraction and cDNA Synthesis

According to the manufacturer's instructions, total RNA was extracted from homogenized rats' aorta tissue with Trizol reagent (Yekta Tajhiz Azma Co., Iran). The quantity of the extracted RNA was determined by NanoDrop (ND1000; Thermo Fisher, Wilmington, Delaware, USA); after identification of 18S and 28S isolated bands on a 2% agarose gel, its yield and quality were validated. Based on the manufacturer's protocol, the cDNA synthesis kit (Pars Tous, Iran) was applied to produce first-strand cDNA with a random hexamer primer [39, 40].

#### 2.6.2. Quantitative Real-Time Polymerase Chain Reaction (QRT-PCR)

QRT-PCR analysis was carried out using the SYBR Green PCR Master Mix (Ampliqon, Denmark) using a Light Cycler System (Roche Diagnostics, Mannheim, Germany) to measure eNOS, LOX-1, and ICAM-1 gene expression; for normalization, beta-actin was used as a housekeeping gene. Primer sequences were designed using data from the National Center for Biotechnology Information database and Beacon Designer software, version 7 (Premier Biosoft International, Palo Alto, California, United States). The primer sequences of the target genes are presented in Table 2. Relative gene expression (fold changes) was calculated using the ΔΔCT method [26, 41].

### 2.7. Statistical Analysis

The GraphPad Prism (version 9.5.1) was used to analyze the data and produce graphs. The Shapiro–Wilk test was used to assess the normality of data, and Bartlett's test for the homogeneity of variances. Normally distributed data was analyzed using analysis of variance (ANOVA) and Tukey's or LSD post hoc tests (if the homogeneity of variance was assumed) and Dunnett's T3 or unpaired *t* with Welch's correction (if the homogeneity of variance was not considered) and expressed as mean ± SD. Nonparametric data was analyzed using the Kruskal–Wallis test with Dunn's multiple comparisons test and expressed as a median with an interquartile range. The level of statistical significance was set at *p* < 0.05.

## 3. Results

### 3.1. Blood Biochemical Parameters

**Figure 2:**
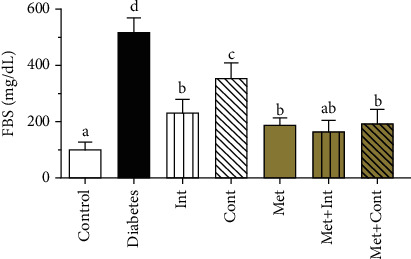
Comparison of the fasting blood sugar (FBS) among different study groups. Means with different letters significantly differ at *α* = 0.05 (*n* = 6 in each group). Statistical analyses were made using the one-way ANOVA followed by Tukey's test. Data are presented as mean ± SD.

In the diabetic group, FBS increased significantly compared to the control group (*p* < 0.05). In all treatment groups, blood glucose decreased significantly compared to the diabetic group (*p* < 0.05). In the Int, Met+Int, Met+Cont, and Met, a significant reduction of FBS was observed compared to the Cont group (*p* < 0.05). As shown in Figure 2, the best response to treatment was observed in the Met+Int group compared to other groups, and there were no significant differences from the control group (Figure 2).

Table 3 demonstrates the results of lipid profile assessment in experimental groups.

Serum levels of TG, TC, and LDL in the diabetes group showed a significant rise compared to the control group (*p* < 0.05), while HDL was increased (*p* = 0.101). In both exercises, Met and the combination of metformin exercise groups TG, TC, and LDL significantly reduced compared to the diabetic rats (*p* < 0.05). At the same time, HDL levels were significantly increased in all treated groups (*p* < 0.05). In addition, the Int group demonstrated a more significant reduction in serum TG levels compared to the Cont group (*p* < 0.05). Met+Int and Met+Cont groups showed a significant decrease in LDL levels compared to the Int and Cont groups (*p* < 0.05).

Compared to the control group, the diabetes group had a higher ratio of TG/HDL, TC/HDL, and LDL/HDL (*p* < 0.05). A significant decrease in these three ratios was observed in the treatment groups compared to the diabetes group (*p* < 0.05).

### 3.2. Oxidative Stress Markers

**Figure 3:**
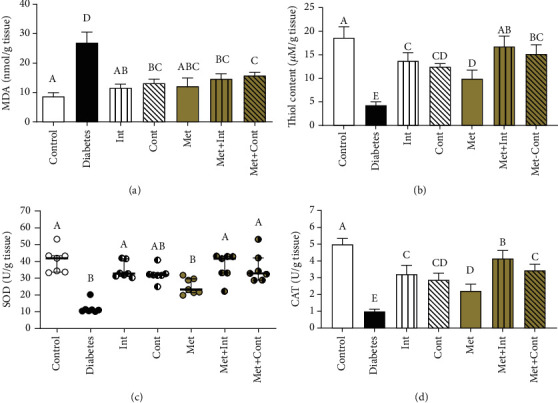
Comparison of oxidative stress parameters among different groups of study. (a) MDA levels, (b) Thiol content, (c) SOD activity, and (d) CAT activity in the aorta tissues. Means not sharing the same letters are significantly different by one-way ANOVA and Tukey's test (A, B, and D) or Kruskal–Wallis with Dunn's test (C) at *α* = 0.05 (*n* = 7 in each group). Data presented as mean ± SD (A, B, and D) or median with interquartile range (C).

In the aortic tissue of the diabetic group, the level of MDA revealed a noticeable increase compared to the control group (*p* < 0.05). In addition, as compared to the diabetes group, there was a significant reduction in tissue MDA levels in all treatment groups (*p* < 0.05) (Figure 3(a)). There were no significant differences between the different types of exercise in the MDA levels (*p* > 0.05).

Thiol levels were significantly lower in the diabetes group than in the control group (*p* < 0.05). Diabetic rats who received metformin treatment, exercise, or their combination showed a significant increase in thiol content (*p* < 0.05). There was no significant difference between the Int or Cont training, but thiol content in the Int group was significantly higher than in the Met group (*p* < 0.05). In the combination groups, the Met+Int group has a greater thiol level than the Int, Cont, and Met groups (*p* < 0.05) (Figure 3(b)).

The results demonstrated that SOD activity in the diabetes group had a significant reduction compared to the control group (*p* < 0.05). There is a considerable augmentation in the level of SOD activity in the treatment groups (except the Met group) compared to the diabetes group (*p* < 0.05). In the exercise groups, SOD activity was significantly higher than the Met group (*p* < 0.05) (Figure 3).

The diabetes group's CAT activity was much lower than the control group (*p* < 0.05). Also, the elevation in CAT activity in the treatment groups was markedly higher than in the diabetes group (*p* < 0.05). In the Met+Int and Met+Cont groups, higher enzyme activity levels were observed compared to the Met group (*p* < 0.05). Met+Int group had a higher level of CAT activity than other groups of intervention (*p* < 0.05) (Figure 3(d)).

### 3.3. Evaluation of Gene Expression

**Figure 4:**
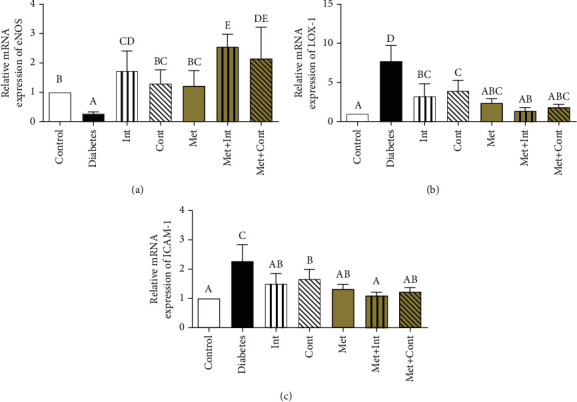
Comparison of the mRNA expression of (a) eNOS, (b) LOX-1, and (c) ICAM-1 among different groups of study. Means not sharing the same letters are significantly different by one-way ANOVA and LSD (eNOS) or Tukey's test (LOX-1 and ICAM-1) at *α* = 0.05 (*n* = 6 in each group). Data are presented as mean ± SD.

eNOS gene expression was downregulated in the diabetes group compared to the control group (*p* < 0.05). The expression of this gene has been enhanced in all treatment groups compared to the diabetes group (*p* < 0.05). Notably, the expression of the eNOS gene in the Met+Int group revealed a more remarkable improvement than the Int, Cont, and Met groups (*p* < 0.05). In the Met+Cont group, the expression of eNOS also increased compared to the Cont and Met groups (*p* < 0.05) (Figure 4(a)).

Upregulation of the LOX-1 gene was detected in the diabetic group's aorta compared to the control group (*p* < 0.05). Compared to the diabetes group, the treatment groups demonstrated a significant reduction in the expression of this gene (*p* < 0.05). Also, the combined exercise groups showed a greater reduction in gene expression than the exercise groups (*p* < 0.05) (Figure 4(b)).

In the diabetes group, ICAM-1 gene expression was upregulated than the control group (*p* < 0.05). The expression of the indicated gene was lower in the intervention groups than in the diabetes group (*p* < 0.05). The decrement in ICAM-1 gene expression is more conspicuous in the Met+Int and Met+Cont groups compared to the Cont group (*p* < 0.05) (Figure 4(c)).

## 4. Discussion

The current study found that interval and Cont, especially when combined with medication, can control blood glucose and dyslipidemia and, as a result, reduce vascular damage by lowering oxidative stress and endothelial inflammation. Of course, there is some paradoxical evidence about the effect of metformin in combination with exercise training in some studies [42, 43].

As our results showed, exercise alone lowered blood glucose levels in diabetic rats, although not as much as the combination and metformin groups. The effect of Int in this reduction process was better than that of Cont. Furthermore, diabetic rats who received metformin in addition to exercise had a better response than the other groups. Previous experimental and clinical investigations have demonstrated the effectiveness of interval and continuous in lowering blood glucose levels [44, 45]. Meanwhile, the benefits of metformin as the preferred drug for the treatment of diabetes and hyperglycemia have been demonstrated to improve biological endothelial function and reduce the risk of cardiovascular disease [21]. MICT has been shown to perform better than HIIT in lowering blood glucose after 8 weeks of training (30 min/day, 5 days/week) in male rats with T2DM who received STZ and a high-fat diet [46]. Controlling blood glucose is one of the most important aspects of preventing vascular complications of diabetes by enhancing oxidant-antioxidant balance and inhibiting some degree of inflammation [44]. Exercise can improve hyperglycemia in several pathways, according to the current and previous studies: boosting insulin sensitivity, especially in skeletal cells, as well as increasing GLUT-4 expression and activity, raising PK-C, which improves the function of insulin-dependent glucose transporters, lowering adipocyte insulin resistance, inhibiting TNF-*α*, a critical factor in inflammation and insulin resistance, also exercise has a protective effect on pancreatic beta cells and stimulating them to secrete more insulin [47, 48].

However, different training protocols and the time gap between training and blood glucose testing and evaluation on human or animal samples may cause discrepancies in some results.

According to our findings, combining exercise and metformin improved the lipid profile. Compared to the diabetic group, both the two types of exercise and the combination groups exhibited a substantial improvement in TG, TC, LDL, and HDL levels as well as in lipid ratios (atherogenic indices). In so far as in the Int and both combination groups, the difference in the level of blood TGs with the control group was not significant, which may indicate the better performance of exercise along with metformin taking, as well as the better performance of Int compared to Cont. By reducing insulin resistance, exercise improves lipid metabolism and inhibits inflammatory pathways [48]. Endurance exercise training (30 min/day, 4 days/week, 8 weeks) in T2DM rats enhanced fatty acid metabolism and attenuated hyperlipidemia independently of blood glucose levels [49].

In rats fed an atherosclerosis-inducing diet, exercise lowered plasma levels of TC, TG, and LDL while increasing HDL [20]. The ratio of TC/HDL and LDL/HDL in male healthy Wistar rats within 4 weeks of the exercise was reduced compared to the control group that did not perform an exercise [50]. Running on the treadmill for both preventive and therapeutic purposes reduced the ratio of TC/HDL and LDL/HDL in a study investigating the effects of preventive and therapeutic exercise on aged and obese rats fed a high-fat diet for 6 weeks [51]. However, a previous study demonstrated HIIT (variant duration, 6 days/week, 10 weeks) reduced LDL and blood cholesterol levels, whereas MICT (60 min/day, 6 days/week, 10 weeks) had no effect or had less effectiveness on lipid profile variables in diabetic subjects and rats after exercise intervention [45, 52]. Also, in one trial, people with T2DM were trained at intervals and continuously for 12 weeks (30-60 min/day, 5 days/week). Still, serum cholesterol TG, LDL, and HDL did not alter considerably [53]. As a consequence of rising HDL, LDL is eliminated from the bloodstream more quickly. Reducing LDL limits foam cell production decreases inflammatory cytokines, and diminishes inflammation [12]. Here also, discrepancies in animal or human models, as well as differences in exercise training programs, could explain the disparity in outcomes.

Our data showed an increase in MDA levels and a decrease in thiol amount, SOD, and CAT activity in diabetic aortic tissue compared to the control group. The oxidative stress factors in the aorta were reduced after 6 weeks of regular treadmill running. Elevated levels of the MDA and reduction of the antioxidant enzyme defense system in diabetes are essential elements in oxidative stress and atherosclerotic inflammation [9]. Here, we observed that the combined group of Met+Int had more comparable effective results in alleviating the balance of oxidant and antioxidant parameters in diabetic animals. The significant difference between the combined and metformin groups indicates that exercise and metformin synergistically affect the oxidant-antioxidant balance. Oxidative stress enhances ROS generation of free intracellular fatty acids by inactivating anti-atherosclerotic enzymes such as eNOS and prostacyclin synthase [9]. Oxidative alterations and hyperlipidemia in diabetes facilitate ox-LDL formation, which, together with inhibition of the release of endothelium-derived relaxation factor (EDRF), finally leads to endothelial dysfunction, inflammation, and vascular injury [9, 54]. Previous findings indicate the role of different types of exercise in the modulation of oxidative balance. Obese rats with a high-fat diet and patients with diabetes and cardiometabolic diseases showed better antioxidant status following Int than continuous training (30-50 min/day, 3–5 days/week, 8–12 weeks) [55–57].

Metformin also can lower ROS in the endothelial cells and relieve oxidative stress by enhancing SOD and CAT activity and decreasing LOX-1 expression [21]. Exercise boosts antioxidant enzymes, which help remove ROS [20, 54].

NO generated in endothelial cells by eNOS regulates vascular tone and inhibits platelet aggregation, vascular proliferation, and monocyte adherence to vascular wall cells. Improving antioxidant status enhances NO bioavailability, critical in preventing endothelial damage [12]. During physical activity, increased shear stress stimulates better eNOS modulation [58]. Exercise-induced relaxation of the collateral coronary arteries has been linked to increased expression of eNOS mRNA and its protein in healthy dogs (120 min/day, 10 days) and miniature swine (85 min/day, 16–20 weeks) [59, 60]. Therefore, the occurrence of endothelial disorders in changes in its amount is not far from the mind [54]. Our findings demonstrated less expression of eNOS in diabetic animals than in the control. The Int group showed a better response than the Cont ones in increasing the expression of eNOS mRNA. Exercise training, but not food restriction, prevents endothelial dysfunction in noninsulin-dependent DM (NIDDM) rats, presumably due to the exercise-induced increase in the production of NO in Otsuka Long–Evans Tokushima fatty (OLETF) rat, a model of spontaneous NIDDM [61]. Similar to our findings, one study of T2DM patients has indicated that Int (30-40 min/day, 3 days/week, 12 weeks) enhanced NO bioavailability significantly more than continuous training (30–40 min/day, 3 days/week, 12 weeks) [55]. Aerobic activity (60 min/day, 5 days/week, 6 weeks) at various intensities for 6 weeks has also been reported to augment eNOS mRNA, eNOS activity, and levels of NO product (nitrite and nitrate) in male diabetic rats. Higher intensity exercise leads to higher shear stress levels and increases eNOS [62]. Metformin has also been proven to improve NO bioavailability and eNOS activity in diabetic rats [63]. Independent-glucose effects of metformin on endothelial dysfunction are not precisely identified, but one study indicates that metformin's therapeutic effects may be related to enhanced eNOS coupling, improved bioavailable NO, (NO/cytotoxic peroxynitrite [ONOO−]) balance, lowered oxidase activity, and other mechanisms beyond glucose regulation that affect endothelial function [64]. Previous reports indicate that Int improves antioxidant status and provides better NO availability than continuous training (40–47 min/day, 3 days/week, 16 weeks) [55, 62, 65]. Meanwhile, the beneficial effects of exercise training in preventing the impairment of endothelium-derived relaxing and hyperpolarizing factors (EDRF and EDHF) in T2DM rats have been attributed to the improvement of hyperglycemia and insulin resistance and increased NO production due to its effect [66]. So, this point can justify our achievements regarding the better effect of the combination of exercise and metformin on the upregulation of the level of eNOS gene expression.

Oxidative damage to the vascular endothelium caused by elevated LOX-1 protein significantly contributes to the development of atherosclerosis; thus, decreased LOX-1 expression may be beneficial in reducing diabetic complications such as atherosclerosis and cardiovascular disease [4]. The present data demonstrated that the expression of the LOX-1 gene is much higher in the diabetes group than in the control group. Studies show that hyperglycemia causes LOX-1 overexpression, and inhibition of LOX-1 by metformin reduces ox-LDL formation [21, 67]. Our interventions indicated that the combined groups of exercise and metformin experienced a more significant decrease in the expression LOX-1 than the exercise-alone groups to the extent that the difference between the combined group and control ones was insignificant. Following our findings, in experiments using animals prone to atherosclerosis, 6–8 weeks of aerobic exercise (30 and 60 min/day, 5 days/week) reduced LOX-1 expression in the common carotid arteries endothelium [18, 68]. Therefore, metformin and exercise appear to affect the reduction of LOX-1 expression synergistically. Atherosclerosis, as one of diabetes's cardiovascular consequences, is a chronic condition caused by inflammatory mechanisms such as leukocyte adhesion. Leukocyte adhesion is regulated by the proliferation of adhesive molecules, including ICAM-1 and VCAM-1 [15]. During hyperglycemia-induced oxidative stress, nuclear factor-kB (NF-*κ*B) activation results from ox-LDL binding to the LOX-1 receptor. NF-*κ*B stimulates inflammatory responses and lipid deposition in arteries by increasing the expression of adhesion molecules.

On the other hand, metformin reduces inflammatory processes by suppressing the inflammatory molecules responsible for leukocyte adhesion, such as ICAM-1 and VCAM-1, and reducing the uptake of ox-LDL by endothelial cells [21]. The current study found that diabetes increased ICAM-1 gene expression in animals compared to the control group, corroborated by previous similar studies [44, 69]. It also appeared that exercise and metformin can diminish ICAM-1 gene expression in diabetic animals. This effect was considerably more prominent in both combined groups of metformin and exercise, especially in the Met+Int groups than in the exercise-alone groups. ICAM-1 expression was lowered by 8 weeks of treadmill training (60 min/day, 5 days/week) in diabetic rats [44] and swimming (60 min/day, 5 days/week) in rats predisposed to atherosclerosis [20]. Exercise and metformin appear to favor the process of reducing vascular inflammation in diabetes, and our findings suggest that Int has a better effect on lowering proinflammatory processes. Differences between our findings and those of other experiments could be attributed to the type of underlying medical condition, the type, severity, and timing of the exercise program. Also, other factors, such as the tissue used, the serum level or expression of the genes in question, and the interval after the intervention intended to investigate, should not be overlooked. Per these cases, some studies suggest that the time and intensity of training may be effective in determining the extent of its impact on endothelial activity [12, 54]. Finally, a study of healthy men showed that both interval and continuous training significantly improved endothelial function [70].

Exercise can lower ROS and oxidative stress by improving hyperglycemia and lipid profile. So, LOX-1, elevated due to hyperglycemic condition, was downregulated following exercise. Exercise has lowered ICAM-1 level in endothelial cells, inhibiting inflammatory cell adhesion and leukocyte attachment to vessel walls and suppressing inflammatory processes. On the other hand, strengthening the antioxidant capacity has improved vascular function by enhancing the expression of eNOS and NO bioavailability. Ultimately, preventing the deposition of lipids and inflammatory factors improved endothelial damage, vascular inflammation, and atherosclerosis. It seems that in our study, the combined Met+Int group has shown the best effect in preventing vascular endothelial damage due to its high capacity to reduce blood glucose, followed by the reduction of oxidative stress.

Overall, based on similar previous studies, interval's superiority in boosting vascular function and preventing endothelial injuries over Cont is owing to its more substantial impact on cardiovascular disease risk factors, insulin resistance, oxidative stress, and inflammation. Although, due to some of the different findings, more research in this area is needed.

### 4.1. Limitations

Due to insufficient aortic tissue representative as a vascular bed, it was impossible to carry out further investigations, including protein measurement of inflammatory parameters and assessing vascular function parameters to support the molecular findings. In addition, histological assessments are valuable for localizing the eNOS expression in the aorta. It is suggested that these cases be taken into consideration in future studies.

Also, as a limitation, the animals in the control group were not assessed for their ability to exercise training. Still, the diabetic animals were evaluated before being randomly assigned to different groups.

On the other hand, although the STZ-induced diabetes model is not purely a T2DM and does not mimic all of the features of this type of disease, it still is the more common model in experimental investigations.

### 4.2. Conclusion

Although some previous studies have investigated and confirmed the positive effects of exercise on metabolic disorders, the new approach of this study is the combination of exercise and metformin, which can be generalized and implemented in the clinic. The findings of our study showed that a combination of exercise, especially Int, and medication may help to manage diabetes better. Physical activity, especially in its interval form combined with metformin, seems to be effective in preventing diabetes-induced functional fluctuation in endothelial biomarkers. This ameliorative effect could be exerted by reducing hyperglycemia, hyperlipidemia, oxidative stress, and inflammation in the course of diabetes.

## Figures and Tables

**Table 1:** Treatment protocols in different groups of rats.

**Groups (** **n** = 7**)**	**Treatment protocols**
Group I (control)	Sodium chloride (0.9%)
Group II (diabetes)	Sodium chloride (0.9%)
Group III (Int)	Interval training
Group IV (Cont)	Continuous training
Group V (Met+Int)	Metformin (300 mg/kg)+Int
Group VI (Met+Cont)	Metformin (300 mg/kg)+Cont
Group VII (Met)	Metformin (300 mg/kg)

**Table 2:** Primer designed for semiquantitative real-time polymerase chain reaction.

**Gene**	**Primer sequence**
*eNOS*	F 5′-GCC TGA GCA GCA CAA GAG-3′ R 5′-CTG TCT GTG TTA CTG GAT TCC TTC-3′
*LOX-1*	F 5′-ATT GTA CAG CAG ACA CAG TTA CTC-3′ R 5′-GTT CCC TCT TTG ATT CTT GTG AAG-3′
*ICAM-1*	F 5′-TCT TGC GAA GAC GAG ACC CTC-3′ R 5′-GCT CTG GGA ACG AAT ACA CAG-3′
*β*-*Actin*	F 5′-AAC CCT AAG GCC AAC CGT G-3′ R 3′-TAC GTA CAT GGC TGG GGT GT-3′

**Table 3:** Serum lipid profile.

**Parameter (mg/dl)/group**	**TG** ^∗∗^	**TC** ^∗∗^	**LDL** ^∗^	**HDL** ^∗∗∗^	**TG/HDL** ^∗∗∗^	**TC/HDL** ^∗∗∗^	**LDL/HDL** ^∗∗∗^
Control	42 ± 8.83 a	70.2 ± 5.3 a	23.8 ± 3.5 b	37.9 ± 8.2 abc	1.2 ± 0.41 c	1.9 ± 0.25 bc	0.7 ± 0.20 ab
Diabetes	114.3 ± 13.0 d	99.8 ± 6.58 b	49.0 ± 7.9 a	28.0 ± 10.6 c	4.7 ± 1.8 a	4.2 ± 1.9 a	2.2 ± 1.57 a
Int	45.3 ± 4.67 ab	74.5 ± 8.98 a	24.2 ± 3.7 b	42.3 ± 10.6 ab	1.2 ± 0.22 c	1.9 ± 0.30 bcd	0.6 ± 0.26 ab
Cont	70.5 ± 16.55 c	78.8 ± 3.87 a	25.8 ± 4.2 b	38.9 ± 3.93 bc	1.8 ± 0.38 b	2.0 ± 0.17 b	0.7 ± 0.17 a
Met	65.7 ± 18.86 bc	77.5 ± 8.92 a	19.2 ± 2.3 bc	45.2 ± 8.6 ab	1.5 ± 0.48 bc	1.7 ± 0.19 cd	0.4 ± 0.13 bc
Met+Int	49.3 ± 7.58 abc	72.0 ± 4.05 a	16.0 ± 1.4 c	46.1 ± 2.11 a	1.1 ± 0.14 c	1.6 ± 0.045 d	0.4 ± 0.03 c
Met+Cont	63.7 ± 12.48 abc	74.5 ± 5.68 a	16.3 ± 1.5 c	45.4 ± 3.8 a	1.4 ± 0.22 bc	1.6 ± 0.046 cd	0.4 ± 0.04 c

*Note*: Means with no letter in common in each column are significantly different by one-way ANOVA and Dunnett's T3 (^∗^), Tukey's (^∗∗^), or unpaired *t* with Welch's correction (^∗∗∗^) post hoc test at *α* = 5% (*n* = 6/group).

Abbreviations: HDL, high-density lipoprotein; LDL, low-density lipoprotein; TC, total cholesterol; TG, triglyceride.

## Data Availability

The data supporting this study's findings are available from the corresponding author upon reasonable request.

## Nomenclature

CAT: catalase
Cont: continuous training
DM: diabetes mellitus
eNOS: endothelial nitric oxide synthase
FBG: fasting blood glucose
GPx: glutathione peroxidase
HDL: high-density lipoprotein
ICAM-1: intercellular adhesion molecule-1
Int: interval training
LDL: low-density lipoprotein
LOX-1: lectin-like oxidized low-density lipoprotein receptor-1
MDA: malondialdehyde
MET: metformin
NO: nitric oxide
ox-LDL: oxidized low-density lipoprotein
ROS: reactive oxygen species
SOD: superoxide dismutase
STZ: streptozotocin
TC: total cholesterol
TG: triglyceride
VCAM-1: vascular cell adhesion molecule-1
